# Supplementary figures and images for: Moving towards a person-centred HIV care cascade: An exploration of potential biases and errors in routine data in South Africa
Source: PLOS Glob Public Health. 2024 Jun 6;4(6):e0002509. doi: 10.1371/journal.pgph.0002509 (PMC11156390; doi:10.1371/journal.pgph.0002509)

**Supplementary Figure 1:** Flow chart of individuals included in the analysis


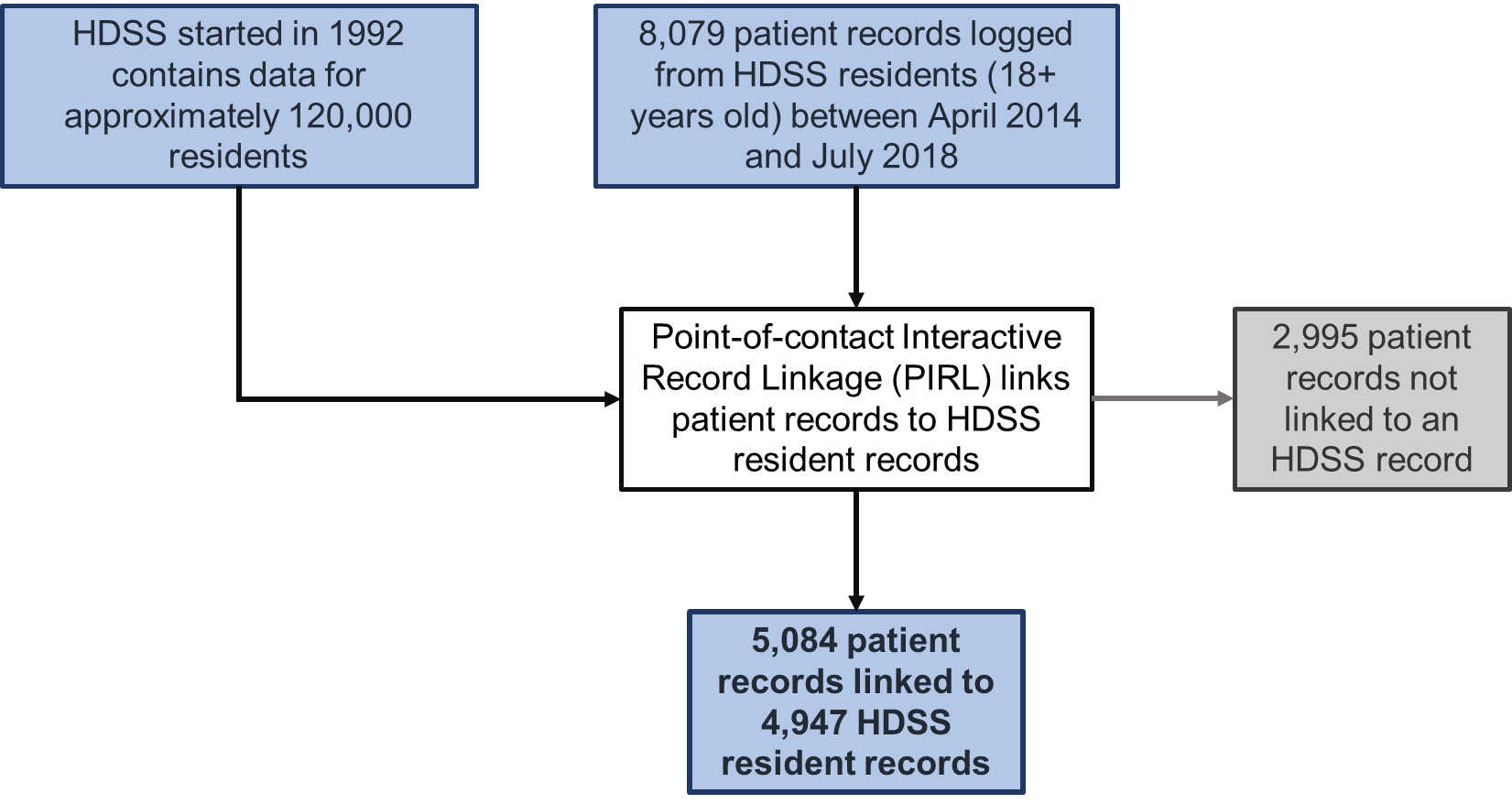

Supplement: S1 Fig — (DOCX) [file pgph.0002509.s002.docx]
