# Supplementary material for: Moving towards a person-centred HIV care cascade: An exploration of potential biases and errors in routine data in South Africa
Source: PLOS Glob Public Health. 2024 Jun 6;4(6):e0002509. doi: 10.1371/journal.pgph.0002509 (PMC11156390; doi:10.1371/journal.pgph.0002509)
Supplement: S2 Fig — (DOCX) [file pgph.0002509.s003.docx]

**Supplementary Figure 2:** Proportion of sequence time spent in each state on average by age


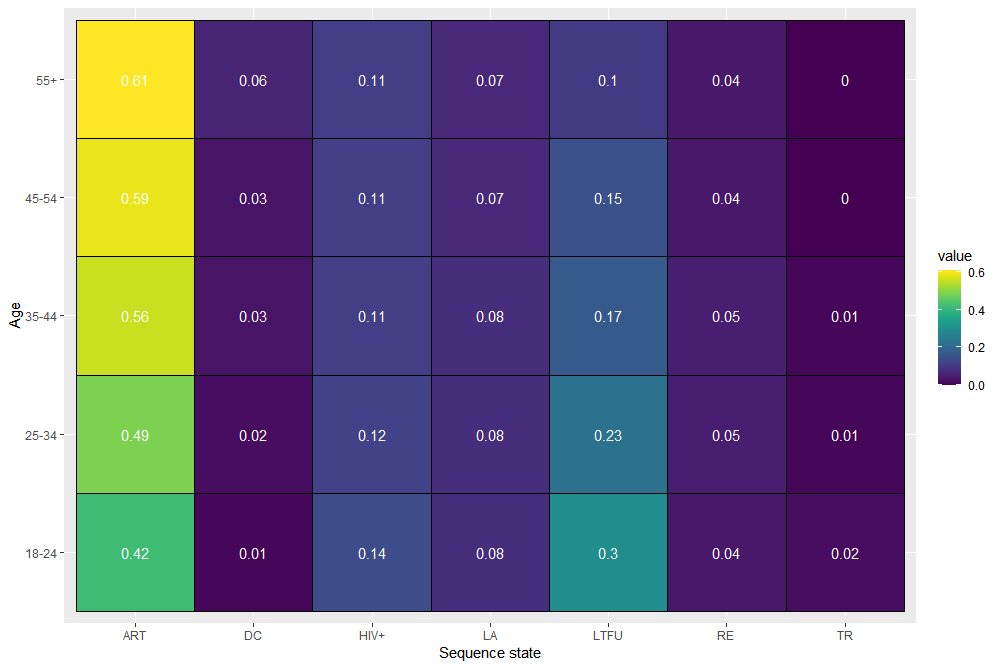


**Note:** ART – On ART, DC – Deceased, HIV+ – Known HIV+ but not on ART, LA – Late, LTFU – Lost to follow-up, RE – Reengaged, TR – Transferred.

**Test for trend:**

| Sequence state | ART | DC | HIV+ | LA | LTFU | RE | TR |
| --- | --- | --- | --- | --- | --- | --- | --- |
| p-value | <0.001 | <0.001 | <0.001 | <0.001 | <0.001 | 0.8108 | <0.001 |
