# Supplementary material for: Moving towards a person-centred HIV care cascade: An exploration of potential biases and errors in routine data in South Africa
Source: PLOS Glob Public Health. 2024 Jun 6;4(6):e0002509. doi: 10.1371/journal.pgph.0002509 (PMC11156390; doi:10.1371/journal.pgph.0002509)
Supplement: Attachment — Submitted filename: Moving towards person centered care NK.docx [file pgph.0002509.s008.docx]

**Important paper that addresses a knowledge gap in estimation of PLHIV in care, patterns and pathways of engagement/disengagement into and out of care**

Study sought to explore potential biases and errors in estimation of PLHIV in care based on reconciliation or a lack thereof, in HDSS vs. PIRL – it assessed for individuals who had had multiple testing for HIV, been **linked to more than one record, or LTFU**

Results section
Line 159: Include a flow chart/diagram from HDSS to PIRL - showing study participants included/excluded based on criteria referred to, (un)/linked to unique identifiers and those included in the analysis

Line 165: Would be good to profile these 137 PLHIV with multiple patient records (social and clinical and demographics). Even better would be a follow-up study to understand reasons for their transfers to contextualise these findings (although this wasn’t the aim of the study).

Lines 169-170: Where are the 226 coming from? Not clear which denominator of the prior listed numbers authors are referring to and text seems fragmented from rest – would be good to make clear how it links to prior text or other section of paper.

Lines 172-173: Does this mean these patients were transferred without any documentation or is it possible that their linking identifiers were somehow not captured in the system? Or could it be that the transferring facilities chose not to issue their clinical data/files because the patients were being transferred to the new facilities as new to ART care? Do we know if these weren’t new PLHIV initiating ART or re-engaging in care after long periods of LTFU?

Lines 178-181Interesting that the majority of patients who had had multiple testing had transferred once. Does this mean that there was no association between multiple testing and multiple linkages?

Lines 200-210: Is it possible to present a trend test p-value for each of the categories to highlight the significance of change in proportion at each of the categorical levels?

Lines 237: Ages 35-54 left out?

Discussion

Line 186: How does the 32.7% statistic of not initiating same day ART in your study compare with the national statistics. Are there reasons authors think uptake of this recommendation has lagged?

The discussion is silent about figure 1 findings of patient care pathways. Aren’t these worth mentioning?

Similarly, not much is mentioned about factors associated with belonging to any of the three engagement clusters.

From the study findings are there any recommendations for HIV program managers and policy makers to consider so more PLHIV are enrolled in the Early ART/Stable engagement cluster?
