# Supplementary material for: Moving towards a person-centred HIV care cascade: An exploration of potential biases and errors in routine data in South Africa
Source: PLOS Glob Public Health. 2024 Jun 6;4(6):e0002509. doi: 10.1371/journal.pgph.0002509 (PMC11156390; doi:10.1371/journal.pgph.0002509)
Supplement: Attachment — Submitted filename: Reviewer comments_PLOS_final.docx [file pgph.0002509.s009.docx]

December 20, 2023

Rashmi Josephine Rodrigues, M.D., Ph.D.

Academic Editor

PLOS Global Public Health

RE: Re-submission of the Manuscript PGPH-D-23-01814

Dear Editor,

Thank you very much for reviewing our manuscript. We greatly appreciate the editor’s and reviewers’ comments and suggestions. We have taken these into account and revised the manuscript accordingly.

We have attached a manuscript with the changes highlighted as well as a clean version.

Please find attached a point-by-point response to the reviewers’ concerns. We hope that you find these responses satisfactory and look forward to hearing back from you soon.

Sincerely,

Dr David Etoori

(On behalf of the authors)

**EDITOR:**

**1. Kindly respond to all the comments from reviewers.**

**2. the primary objective of your study says**

**'we use a novel approach linking public health facilities offering ART in rural north-eastern South Africa with population- based data, to evaluate routine data for the potential biases that might accrue when utilising it to measure programmatic indicators and progress towards national and global targets.'**

**a. The novel approach that you refer to here- is this what you developed for the purpose of the study or is this something that the health system has developed to link public health facilities?**

This was developed for research purposes not by the health system. We have now clarified this in the manuscript. (Line 113)

**b. what are the programmatic indicators that you are referring to?**

In response to your question, we have added more information of the indicators we are referring to. These include numbers tested, new HIV diagnoses, and numbers engaged. (Lines 115-116)

**3. How much of missing data was found and how was missing data handled.**

Missing data on variables analysed was low ranging between 4-7%. We conducted a complete case analysis and did not utilise any missing data techniques in our analyses. We have made this explicit in the methods section. (Lines 154-155)

**4. Were there any specific assumptions made for the purpose of analysis?**

We assumed that individuals were taking their ART prescriptions as recommended as we had no adherence data. We have now added this to the methods section and to our limitations. (Lines 140-141, 332-334)

**5. How does identification of clusters help achieve your objective of identifying potential biases?**

This is a good question. Historically, HIV programmes have used aggregate data which lacks the nuance of individual level data. The cluster analysis demonstrates the multiple ways that individuals engage with care showing that this does not follow a linear path. This is something that would not be possible with aggregate data. The clusters could also potentially assist programmes in estimating how many individuals within their programmes might have similar HIV care engagement profiles. Following on from your question, we have now added this to the discussion. (Lines 291-296)

**5. Is logistic regression and the OR the best approach to identify associated factors especially when longitudinal data was available. What was the outcome variable used in this analysis?**

Yes, in this case it would be the most appropriate approach. We aimed to understand factors associated with membership in the three engagement clusters we identified. The outcome variable was a binary variable coded as 1 if an individual was a member of a given cluster and 0 otherwise. We mention this in the methods and have added more information to make this clearer. (Lines 149-150)

**6. Are there any limitations with regards to the analytical/ statistical methods used.**

Optimal matching has some limitations. We have added this to the limitations section of the discussion. (Lines 334-335)

**Reviewer #1:**

**The authors address an important issue in HIV programmes - the transition from aggregated to person-centered monitoring on the HIV care cascade is important to improving outcomes. The authors are also well-positioned to address the topic because of the PIRL Database.**

We thank the reviewer for their positive comments.

**While the authors have access to high quality data, the manuscript could be improved by (1) being clear on terminology so that the reader is able to follow, and (2) better alignment of the problem statement/rationale with results. Regarding the latter point, there needs to be careful though into the results presented and the theme of potential biases/errors vs. broader/general descriptive analyses that do not add any answers to the research question. The lack of consistency in the use of terms makes it difficult to interpret tables and results.**

By addressing each of the helpful specific comments below we believe we have addressed these issues. **Below are specific comments:

Line 30 – Please clarify – this statement “The switch to routine data will likely introduce some biases and errors.” – the switch to routine data from what?**

We have now made this statement clearer. “The switch from aggregated service level data to person-centred data will likely introduce some biases and errors.” (Lines 30-31)

**Line 46 - a low level of sustained engagement in care 47 (<33%) – considering that significant portion of the abstract speaks to engagement patterns – it will be helpful to understand what “sustained” engagement means. Also, does this differ from “stable engagement” used in line 50.**

Thank-you for highlighting that we used sustained and stable engagement interchangeably. By sustained engagement, we refer to a situation where a patient consistently visits a clinic to collect their ART prescriptions and adheres to guidelines set by clinic staff. We have now changed sustained to stable to avoid any confusion. (Line 47)

**Line 51 – Please clarify what the numbers in the brackets represent…” individuals who initiated ART in 2017 versus 2016 (77.44 (43.33, 138.39)), and under test and treat versus due to CD4 eligibility (1.78 (1.31, 2.42))...”**

Thank you for this point of clarification. We have now specified that these numbers represent 95% confidence intervals throughout the abstract. (Lines 53-60)

**Line 54 – Is there a number missing after WHO Stage? “…with OIs [WHO stage: 1.57 (1.07, 2.30); TB: 2.47 (1.60, 3.80))…”**

WHO stage is compared to ART initiation due to low CD4 count. Although no number is missing, we have now reworded this section to clarify this. (Line 56)

**Line 56 – please clarify that 55+ is in “years”**

Thank you for this suggestion. We have now added years as suggested (Line 58).

**Line 56 – Please clarify the comparison group for this statement to improve clarity “those who initiated ART due to CD4 eligibility” (i.e., compared with who?)**
Thank you for catching this. This was accidentally left in the abstract during final edits. We have now removed it to avoid any confusion. (Lines 58-59)

**Line 77 – Please provide further clarity to this statement – “The expansion in treatment programmes has not been matched by an appropriate growth in data infrastructure, with many programmes still reliant on paper registers.” Please clarify where the setting to which this statement should be inferred. The authors use references 8 and 9, but the stated references do not fully support this generalization.**

This was meant to represent the sub-Saharan African context. We have now added more information to make this explicit. (Line 80)

**Line 83 – consider revising this sentence, there is repetition - and the need to better understand patterns of engagement, disengagement and engagement with HIV services.**

Thank you for catching this. We have now revised the sentence to avoid repetition. (Line 86)

**Line 131 – Authors specify when the cohort follow up ended (This cohort was followed until July 11, 2018). Please clarify when follow up started.**

Follow-up started in April 2014. This has now been clarified. (Line 133)

**Line 165 – “Of the 137 (2.8%) with multiple patient records, 127 (2.6%) had transferred once, 3 (0.1%) had transferred twice, and 4 (0.1%) had re-engaged at the same facility but had been given a new file number” – should the breakdown add to 137 (i.e., 127+3+4)?**

Apologies for this confusion. There were 137 extra patient records linked to 134 unique HDSS residents. We have now changed this to 134 to reflect the unique individuals rather than the extra records. (Lines 169-172)

**Line 167 – do the 133 total transfers refer to those with multiple patient records. If they do, then the text above shows that there were 130 transfers.**

The number is 133. 127 patients transferred once and 3 transferred twice each therefore accounting for 6 total transfers.

**Table 2 – introduces new terms that have not been defined in Table 1 – unstable, ‘prolonged’ LTFU, early ART, stable. Because of this, it is difficult to interpret Table 2.**

These refer to the characteristics of the three identified clusters. We now reference Table 2 after the clusters have been described. We have also edited Table 2 to make this clear. (Lines 181, 190, 237)

**Line 180 – please clarify what numbers in brackets represent - [90/127, 70.9% (62.1-78.6)]**

Thank you for this suggestion. We have now indicated that the numbers refer to 95% confidence intervals throughout the results section. (Lines 192-198)

**Reviewer #2:**

**Important paper that addresses a knowledge gap in estimation of PLHIV in care, patterns and pathways of engagement/disengagement into and out of care
Study sought to explore potential biases and errors in estimation of PLHIV in care based on reconciliation or a lack thereof, in HDSS vs. PIRL – it assessed for individuals who had had multiple testing for HIV, been linked to more than one record, or LTFU.**

We thank the reviewer for their positive comments.

**Results section
Line 159: Include a flow chart/diagram from HDSS to PIRL - showing study participants included/excluded based on criteria referred to, (un)/linked to unique identifiers and those included in the analysis**

We have now added a flow chart to the Supplementary Information file. (Line 172)

**Line 165: Would be good to profile these 137 PLHIV with multiple patient records (social and clinical and demographics). Even better would be a follow-up study to understand reasons for their transfers to contextualize these findings (although this wasn’t the aim of the study).**

Thank you for this suggestion. We have added information for the 134 individuals linked to multiple clinic records. (Lines 174-178). In summary we have reported their median age and baseline CD4 and their sex and ART initiation year distribution.

**Lines 169-170: Where are the 226 coming from? Not clear which denominator of the prior listed numbers authors are referring to and text seems fragmented from rest – would be good to make clear how it links to prior text or other section of paper.**

Thank-you for highlighting this. This was based on information in the patient’s clinic records. We have now stated this in the manuscript. (Line 182)

**Lines 172-173: Does this mean these patients were transferred without any documentation or is it possible that their linking identifiers were somehow not captured in the system? Or could it be that the transferring facilities chose not to issue their clinical data/files because the patients were being transferred to the new facilities as new to ART care? Do we know if these weren’t new PLHIV initiating ART or re-engaging in care after long periods of LTFU?**

It is correct to suggest that they could have been newly diagnosed and that the transferring facility could have chosen not to open a clinic file for them in this situation. We referred to this when stating “they never had a treatment file opened at these facilities.” In response to the reviewer’s comment, we have now been more explicit, stating “(e.g., newly diagnosed who request to start treatment at another facility).” (Lines 186-187)

**Lines 178-181Interesting that the majority of patients who had had multiple testing had transferred once. Does this mean that there was no association between multiple testing and multiple linkages?**

An interesting point. In our analysis multiple linkages were synonymous with transfers as this was the most common way we could identify individuals who had moved facilities. As such, multiple testing is associated with multiple linkages. We have now made this clear in the manuscript. (Lines 194-195)

**Lines 200-210: Is it possible to present a trend test p-value for each of the categories to highlight the significance of change in proportion at each of the categorical levels?**

In response to the reviewer’s comment, we have performed a trend test for the ordered categories (age, CD4, and ART initiation year) and added this to the supplementary information file.

**Lines 237: Ages 35-54 left out?**

Thank you for this suggestion. We have now edited the section as suggested. (Line 253)

**Discussion
Line 186: How does the 32.7% statistic of not initiating same day ART in your study compare with the national statistics. Are there reasons authors think uptake of this recommendation has lagged?**

Our estimate is similar to other studies from this setting. Early ART initiation could have lagged for several reasons. For example, individuals might not be ready to start, TB co-infection is also common in this setting and delayed ART is recommended in these cases as well as in cases with some opportunistic infections such as cryptococcal meningitis. In response to the reviewers comment we have expanded our discussion to cover this. (Lines 296-299)

**The discussion is silent about figure 1 findings of patient care pathways. Aren’t these worth mentioning?**

Thank you for this suggestion. We now mention these findings in the discussion. (Lines 299-306)

**Similarly, not much is mentioned about factors associated with belonging to any of the three engagement clusters.**

Thank you for this suggestion. We now cover these findings in the discussion. (Lines 291-296, 299-306)

**From the study findings are there any recommendations for HIV program managers and policy makers to consider so more PLHIV are enrolled in the Early ART/Stable engagement cluster?**

Thank you for this suggestion. We have now added some recommendations to the discussion. For example, we recommend that programmes may need to evolve to offer care tailored to individual needs. We also recommend that programmes may need to pay particular attention to younger individuals, pregnant women, individuals with OIs, those with higher CD4 counts at diagnosis, and males as these groups are least likely to remain stable on ART and could benefit from extra interventions. (Lines 304-306, 339-342)
