# Supplementary material for: Moving towards a person-centred HIV care cascade: An exploration of potential biases and errors in routine data in South Africa
Source: PLOS Glob Public Health. 2024 Jun 6;4(6):e0002509. doi: 10.1371/journal.pgph.0002509 (PMC11156390; doi:10.1371/journal.pgph.0002509)
Supplement: Attachment — Submitted filename: Review Comments.docx [file pgph.0002509.s010.docx]

The article presents valuable insights into the biases inherent within routine clinical data used for HIV program monitoring. Its exploration of patient journeys through treatment is timely, given WHO’s emphasis on person-centered data and individual engagement patterns.

1. The introduction outlines the context and challenges in HIV care data management, but the study's specific objective must be clearly stated. A concise statement of the objectives would enhance the reader's understanding.
2. Line 148: Instead of 3 logistic regression model, use a single multinomial regression model. It would improve the precision of the estimates.
3. Line 175, give full form of PMTCT at the first instance. Similarly, give full form of all abbreviations on the first instance.
4. Figure 1, the graphs do not have a title or labels on the x-axis.
5. Lines 167 – 240: To enhance the readability and interpretation of the results, consider organizing the extensive numerical data into well-structured tables.
6. In the logistic regression model for 'Late ART/Unstable' in Table 3. Could you please clarify why age wasn't included as a dependent variable? Is it because age does not have statistical significance. If so, is there any reason for that.
