# Supplementary material for: Moving towards a person-centred HIV care cascade: An exploration of potential biases and errors in routine data in South Africa
Source: PLOS Glob Public Health. 2024 Jun 6;4(6):e0002509. doi: 10.1371/journal.pgph.0002509 (PMC11156390; doi:10.1371/journal.pgph.0002509)
Supplement: Attachment — Submitted filename: Reviewer comments_PLOS_R2.docx [file pgph.0002509.s011.docx]

April 20, 2024

Rashmi Josephine Rodrigues, M.D., Ph.D.

Academic Editor

PLOS Global Public Health

RE: Re-submission of the Manuscript PGPH-D-23-01814-R1

Dear Editor,

Thank you very much for reviewing our manuscript. We greatly appreciate the editor’s and reviewers’ comments and suggestions. We have taken these into account and revised the manuscript accordingly.

We have attached a manuscript with the changes highlighted as well as a clean version.

Please find attached a point-by-point response to the reviewers’ concerns. We hope that you find these responses satisfactory and look forward to hearing back from you soon.

Sincerely,

Dr David Etoori

(On behalf of the authors)

**Reviewer #3:**

**The article presents valuable insights into the biases inherent within routine clinical data used for HIV program monitoring. Its exploration of patient journeys through treatment is timely, given WHO’s emphasis on person-centred data and individual engagement patterns.**

We thank the reviewer for their positive comments and agree that our results are timely and provide an exemplar for person-centred data approaches.

**1. The introduction outlines the context and challenges in HIV care data management, but the study's specific objective must be clearly stated. A concise statement of the objectives would enhance the reader's understanding.**

Thank you for this suggestion. We have now concisely rewritten the objectives of the study. (Lines 121-124)

**2. Line 148: Instead of 3 logistic regression model, use a single multinomial regression model. It would improve the precision of the estimates.**

Thank you for this suggestion. We now present results based on a multinomial logistic regression. We used Late ART/Unstable as the base outcome and compared the other outcomes (Early ART/Unstable and Early ART/Prolonged LTFU) to this outcome (Lines 41; 51-59; 158; 241-251)

**3. Line 175, give full form of PMTCT at the first instance. Similarly, give full form of all abbreviations on the first instance.**

Thank you for this suggestion. We have now added the full form as suggested. (Lines 184-185)

**4. Figure 1, the graphs do not have a title or labels on the x-axis.**

We have now added a label to the x-axis (Figure 1). The Figure title can be found at the end of the document in line with journal submission guidelines (Line 519).

**5. Lines 167 – 240: To enhance the readability and interpretation of the results, consider organizing the extensive numerical data into well-structured tables.**

We agree that well-structured tables would be helpful. We had originally included them as a separate document but have now added them to the end of the manuscript (lines 508-515).

**6. In the logistic regression model for 'Late ART/Unstable' in Table 3. Could you please clarify why age wasn't included as a dependent variable? Is it because age does not have statistical significance. If so, is there any reason for that.**

In the previous analysis, age wasn’t included because it was not statistically significant. With regards to reasons why, I can only speculate. This group will likely include people who have discontinued treatment (more likely for younger participants) and those who have died (more likely for older participants) but whose death has not been reported. This analysis has now been rerun using multinomial logistic regression as you suggested and Late ART/Unstable is used as the base outcome. (Table 3)
